# Synergistic Effects of Unintended Pregnancy and Young Motherhood on Shaking and Smothering of Infants among Caregivers in Nagoya City, Japan

**DOI:** 10.3389/fpubh.2017.00245

**Published:** 2017-09-22

**Authors:** Aya Isumi, Takeo Fujiwara

**Affiliations:** ^1^Department of Global Health Promotion, Tokyo Medical and Dental University, Tokyo, Japan

**Keywords:** shaken baby syndrome, child abuse, shaking, smothering, unintended pregnancy, young parents, prevention

## Abstract

**Background:**

Shaking and smothering in response to infant crying are forms of child abuse that often result in death. Unintended pregnancy and young motherhood are risk factors of such child maltreatment that are often comorbid, few studies have examined their synergistic effect on shaking and smothering of infants. We examined the synergistic effects of unintended pregnancy and young motherhood on shaking and smothering among caregivers of infants in Japan.

**Methods:**

In this retrospective cohort study, a questionnaire was administered to caregivers enrolled for a health check for 3- to 4-month-old infants between October 2013 and February 2014 in Nagoya City, Japan. The questionnaire data were linked to those from pregnancy notification forms registered at municipalities and included information on women’s age and feelings about their pregnancy (*N* = 4,159). Data were analyzed using logistic regression analysis in 2016.

**Results:**

Shaking and smothering of 3- to 4-month-old infants occurred at least once in the past month in 2.0 and 1.5% of cases, respectively. Of all participants, 24.8% reported unintended pregnancy while 7.3% were younger than 25 years old. Infants of young mothers (under 25 years old) with unintended pregnancy were 2.77 [95% confidence interval (CI): 1.15–6.68] and 5.61 (95% CI: 2.40–13.1) times more likely to be shaken and smothered, respectively, than those of older mothers with intended pregnancy. In addition, the odds ratio of young mothers with unintended pregnancy regarding smothering was significantly higher than that of older mothers with unintended pregnancy (odds ratio: 2.12; *p* = 0.02).

**Conclusion:**

Our findings suggest a synergistic effect of unintended pregnancy and young motherhood on smothering. Infants of young mothers with unintended pregnancy are at greater risk of abuse, especially smothering. Prevention strategies are required for young women with unintended pregnancies.

## Introduction

Abusive head trauma (AHT), also known as shaken baby syndrome (SBS), is a severe and often fatal form of infant physical abuse caused by violent shaking and/or impact to the head (1, 2). Previous studies using national databases of child deaths or violent deaths in the United States showed that AHT/SBS accounted for 30.3% of child maltreatment fatalities in children aged under 18 years (3), and 62.5% in children aged under 5 years (4). Similarly, the Japanese Ministry of Health Labor and Welfare reported that 39.3% of deaths due to child abuse among children aged under 18 years in 2013 were caused by AHT (5). Smothering, which is defined as intentionally suffocating a baby with a hand or object such as a cloth, pillow, or plastic wrap (6), is also a form of life-threatening infant abuse in response to crying (7, 8). A UK study of children killed by their parents indicated that 38.0% of these parents confessed to smothering or choking their child (9). In Japan, suffocation without choking, which is considered smothering, accounted for 17.9% of child abuse deaths among children aged under 18 years in 2013 (5). Furthermore, the prevalence of self-reported shaking and smothering at least once in the previous month among Japanese parents of 3- and 4-month infants is 2.4–3.9 and 2.4–2.7%, respectively (10–12). This rate is similar to those reported for other developed countries, such as the Netherlands and the United States (10, 11). Previous studies have identified risk factors of shaking and smothering to develop effective prevention strategies for deaths due to child abuse. Risk factors identified during pregnancy, including first pregnancy (10) and young maternal age (10), are critical for the primary prevention of infant abuse.

Unintended pregnancy, defined as pregnancy that is either mistimed or unwanted at the time of conception, can have adverse effects on the health and well-being of the pregnant woman, her child, partner, and family (13, 14). Importantly, children born to unintended pregnancies are at a greater risk of child maltreatment (13, 15–19). A previous population-based longitudinal study in the United Kingdom found that children registered for child protection services by the age of six were nearly three times more likely to be born to unintended pregnancy than those who were not registered (17). Further, a longitudinal birth-cohort study in the United States showed that mothers with unintended pregnancy were 1.61 and 1.45 times more likely to show psychological aggression and to neglect their children, respectively (19).

The association between unintended pregnancy and child maltreatment has also been observed among Japanese mothers. A survey of mothers of 3- to 18-month-old children showed that women who had had unintended pregnancies were less likely to deny abusing their child (20). Furthermore, recent national data indicated that 54.5% of infants who died due to child maltreatment in fiscal year 2014 in Japan were born to unintended pregnancies (21). However, the association between unintended pregnancy and the shaking and smothering of infants remains unknown.

Unintended pregnancy often coincides with young motherhood (22, 23), which is a major risk factor for shaking and smothering (10). Although data on the comorbidity of young motherhood and unintended pregnancy are limited in Japan, the abortion rate in 2013 was 6.6 per 1,000 females aged 15–19 years versus a birth rate of only 4.4 (24, 25), suggesting that unintended pregnancy among young mothers is not rare. Moreover, U.S. data in 2008 show that unintended pregnancies accounted for 69.3 and 40.2% of all pregnancies by women aged 24 years and younger and those aged 25 years and older, respectively (22). Since younger adults often respond to stressful events, such as unintended pregnancies, with higher levels of anxiety and avoidance compared to older adults (26, 27), younger mothers with unintended pregnancies may be at a higher risk of infant abuse. However, few studies have examined the synergistic effect of both unintended pregnancy and young motherhood on shaking and smothering of infants.

We examined the synergistic effects of unintended pregnancy and young motherhood on shaking and smothering among caregivers of 3- to 4-month-old infants in Japan.

## Materials and Methods

### Study Population

This study targeted primary caregivers (*N* = 4,998), which could include either mothers or fathers, with 3- to 4-month-old infants who were enrolled for a national health check between October 2013 and February 2014 in Nagoya City, the capital of Aichi Prefecture. In 2003, the population of Nagoya City was 2,271,380, with 19,492 births. An anonymous questionnaire with a child ID number was mailed to the target population before, and responses were collected during, the 3- to 4-month health check. A total of 4,666 caregivers completed the questionnaire (response rate 93.4%). The questionnaire data with child ID numbers were linked with those from pregnancy notification forms with maternal ID numbers, which were registered at public health centers in Nagoya City through household ID numbers. In most cases (96.6%), mothers who were registered on pregnancy notification forms answered the questionnaires at the 3- to 4-month health check. In some cases, however, partners or family members of those registered on pregnancy notification forms answered the questionnaires at the 3- to 4-month health check as primary caregivers. Some questionnaire data could not be linked due to translocation of the participants from other municipalities (*N* = 250). Further, those who did not provide answers to the outcome (i.e., shaking, smothering) and exposure (i.e., maternal age, feeling when notified of pregnancy), variables were excluded (*N* = 257). Data from 4,159 caregivers was analyzed.

### Measures

#### Shaking and Smothering

The questionnaire assessed whether respondents had shaken or smothered their infant in the past month. Frequency of shaking behavior was determined with the question: “When your child was crying and making a scene, how many times have you violently shaken your child?” Similarly, frequency of smothering was determined with the question: “How many times have you covered the mouth of your baby with your hands, a cushion, etc., when he/she was crying?” The respondents selected their answer from the following response items: “0 times,” “1 or 2 times,” “3–5 times,” “6–10 times,” and “11 or more times.” These responses were dichotomized based on frequency, with 0 times equated to a “no” response and one or more times equated to a “yes” response.

#### Young Motherhood and Unintended Pregnancy

Pregnancy notification forms included information on maternal age at registration, gestational weeks at registration, birth order, feelings when notified of the pregnancy, and prenatal situation (see below). We defined young mothers as women aged younger than 25 years at pregnancy because (1) late childbearing has been increasing in Japan and teenage mothers represent a very small proportion of the population as well as of our sample, (2) previous studies have indicated women aged 24 years or younger are at a high risk of infant abuse (10), and (3) data from the United States show that women aged 20–24 years have the highest proportion of unintended pregnancies ending in birth (22). Women’s feelings when notified of their pregnancy were assessed with the question: “How did you feel when you found out you were pregnant?” The respondents could select a response from “happy,” “unexpected but happy,” “unexpected and puzzled,” “did not know what to do,” “no feeling,” and “other.” We defined unintended pregnancy as responses of “unexpected but happy,” “unexpected and puzzled,” “did not know what to do,” “no feeling,” or “other.”

#### Covariates

The questionnaire also included questions about postpartum situation, such as perception of the amount of infant crying, subjective socioeconomic status, and postpartum depression. Perception of the amount of infant crying was determined with the question: “Does your baby cry a lot?” Respondents answered using a 5-point Likert scale: 1 = “yes, a lot,” 2 = “yes, relatively,” 3 = “neither yes nor no,” 4 = “no,” and 5 = “not at all.” We categorized these responses into three groups: “not at all” (response 5), “not so much” (responses 3 and 4), and “some extent to a lot” (responses 1 and 2). Current economic situation, used to indicate subjective socioeconomic status, was determined using a 4-point Likert scale: 1 = “stable,” 2 = “able to manage,” 3 = “difficult to manage,” and 4 = “unstable.” We defined economic hardship as responses of “difficult to manage” and “unstable” (responses 3 and 4). Postpartum depression was assessed using the Japanese version of the Edinburgh Postnatal Depression Scale (EPDS) (28). We defined postpartum depression as an EPDS score of 9 or higher based on a previous study examining the reliability and validity of the Japanese version of the EPDS (28). The previous study showed that the internal consistency coefficient (Cronbach’s alpha coefficients) of the total score of 10 items was 0.78, and that the sensitivity (0.75) and specificity (0.93) were highest when the cut-off score was 8 or 9 (28).

We defined late registration as 12 gestational weeks or later. Prenatal information obtained in the pregnancy notification forms included whether patients planned to return to their parents’ home for delivery, had someone who could help, worries or anxiety, and depression. Depression during pregnancy was determined with the binary question: “In the past year, did you experience any of the following symptoms that continue for more than two weeks: ‘inability to sleep,’ ‘feeling irritated/frustrated,’ ‘being emotional,’ or ‘not feeling like doing anything’?”

#### Statistical Analysis

Multiple logistic regression analyses were used to assess the effect of unintended pregnancy and young motherhood separately, and their synergistic effect on shaking and smothering. After estimating a bivariate model, prenatal situation (late registration, birth order, returning to parents’ home for delivery, having someone who can help, worries/anxiety, and depression), as determined in the pregnancy notification forms, was added to Model 1. In addition to covariates in Model 1, Model 2 adjusted for postpartum situation (crying, economic hardship, and depression measured by EPDS), as assessed in the questionnaire at 3- to 4-month health check. All analyses were conducted using Stata/SE v13.1 software in 2016.

## Results

Table 1 summarizes the respondents’ characteristics. Of our respondents, 2.0 and 1.5% reported shaking and smothering at least once in the past month, respectively. Mothers younger than 25 years old (young mothers) accounted for 7.3% of our sample and 24.8% had unintended pregnancies. Further, 70.9% of the samples were mothers aged 25 years and older (older mothers) with intended pregnancy, while 21.8% were older mothers with unintended pregnancy, 4.4% were young mothers with intended pregnancy, and 2.9% were young mothers with unintended pregnancy.

**Table 1:** Characteristics of respondents (*N* = 4,159).

	*N*	%
**Infant abuse**		
Shaking	83	2.0
Smothering	64	1.5
**Unintended pregnancy and maternal age**		
Intended, ≥25 years old	2,948	70.9
Intended, <25 years old	184	4.4
Unintended, ≥25 years old	908	21.8
Unintended, <25 years old	119	2.9
**Maternal age**		
<20	47	1.1
20–24	256	6.2
25–29	1,285	30.9
30–34	1,570	37.7
35–39	863	20.8
≥40	138	3.3
**Feeling at pregnancy**		
Happy	3,132	75.3
Unexpected but happy	793	19.1
Unexpected and puzzled	178	4.3
Did not know what to do	23	0.6
No feeling	9	0.2
Other	24	0.6
**Variables from pregnancy notification forms**		
Late registration (≥12 weeks)		
Yes	380	9.1
No	3,778	90.8
Missing	1	0
Birth order		
First child	2,065	49.7
Subsequent child	2,089	50.2
Missing	5	0.1
Returning to parents’ home for delivery		
Yes	1,281	30.8
No	2,807	67.5
Missing	71	1.7
Having someone who can help		
Yes	3,976	95.6
No	146	3.5
Missing	37	0.9
Worries/anxiety		
Yes	1,457	35.0
No	2,649	63.7
Missing	53	1.3
Depression		
Yes	253	6.1
No	3,885	93.4
Missing	21	0.5
**Variables from the questionnaire at 3- to 4-month health check**		
Crying		
Not at all	2,277	54.8
Not so much	1,262	30.3
Some extent to a lot	610	14.7
Missing	10	0.2
Economic hardship		
Yes	396	9.5
No	3,707	89.1
Missing	56	1.4
Postpartum depression by Edinburgh Postnatal Depression Scale		
≥9	251	6.0
≤8	3,875	93.2
Missing	33	0.8

In our sample, 9.1% of respondents registered their pregnancy at 12 gestational weeks or later and almost half were having their first child. Further, 30.8% indicated that they would return to their parents’ home for delivery. More than 95% reported that they had someone who could help in times of trouble, while 35% had worries or anxiety, and 6.1% were regarded as having depression during pregnancy. Responses to questionnaires at the 3- to 4-month health check indicated that 14.7% perceived their infant as crying “a lot” or “to some extent.” Nearly 10% of respondents reported that their current socioeconomic status was “difficult to manage” and “unstable.” Approximately 6% of caregivers were considered at risk of having postpartum depression [an EPDS score of 9 or more (28)], which is similar to the proportion regarded as having depression during pregnancy.

Table 2 summarizes the odds ratios (ORs) for unintended pregnancy and maternal age on shaking. Independently, unintended pregnancy was significantly associated with shaking even after controlling for postpartum situation in Model 2 (OR: 1.93, 95% CI: 1.20–3.11), although maternal age had no significant effect in the adjusted models (Model 1 and Model 2).

**Table 2:** Odds ratios (OR) for unintended pregnancy and maternal age for shaking in 3- to 4-month-old infants.

		Prevalence of shaking (%)	Crude		Model 1		Model 2	
			OR	95% CI	OR	95% CI	OR	95% CI
Unintended pregnancy	No	1.6	Ref		Ref		Ref	
	Yes	3.2	**2.05**	**1.31–3.20**	**1.93**	**1.21–3.08**	**1.93**	**1.20–3.11**

Maternal age	≥25	1.9	Ref		Ref		Ref	
	<25	3.6	**1.98**	**1.04–3.78**	1.44	0.73–2.83	1.36	0.68–2.72

Late registration	No	1.8	Ref		Ref		Ref	
	Yes	3.7	**2.06**	**1.15–3.69**	1.76	0.96–3.23	1.69	0.91–3.11

Birth order	Subsequent child	1.5	Ref		Ref		Ref	
	First child	2.5	**1.72**	**1.10–2.69**	**1.71**	**1.07–2.73**	1.44	0.89–2.31

Returning to parents’ home for delivery	No	1.9	Ref		Ref		Ref	
	Yes	2.1	1.12	0.70–1.79	1.11	0.69–1.79	1.08	0.67–1.73
	Missing	4.2	2.29	0.70–7.51	1.97	0.58–6.71	2.25	0.65–7.81

Having someone who can help during pregnancy	No	2.1	Ref		Ref		Ref	
	Yes	2.0	0.95	0.30–3.06	1.18	0.36–3.85	1.34	0.40–4.46
	Missing	5.4	2.72	0.44–16.9	3.12	0.48–20.3	3.14	0.47–21.0

Worried/anxious during pregnancy	No	1.8	Ref		Ref		Ref	
	Yes	2.5	1.40	0.90–2.18	1.13	0.71–1.80	1.00	0.62–1.61

Depression during pregnancy	No	2.0	Ref		Ref		Ref	
	Yes	2.4	1.29	0.52–2.79	0.98	0.41–2.31	0.87	0.36–2.10

Crying	Not at all	1.0	Ref				Ref	
	Not so much	2.7	**2.84**	**1.65–4.87**			**2.71**	**1.56–4.70**
	Some extent to a lot	4.4	**4.75**	**2.68–8.40**			**4.17**	**2.32–7.49**

Economic hardship	No	1.9	Ref				Ref	
	Yes	2.3	1.19	0.59–2.40			0.79	0.37–1.68
	Missing	5.4	2.90	0.88–9.50			1.00	0.15–6.41

Postpartum depression by Edinburgh Postnatal Depression Scale score	≤8	1.8	Ref				Ref	
	≥9	4.8	**2.81**	**1.50–5.26**			**2.10**	**1.06–4.14**
	Missing	9.1	**5.60**	**1.67–18.8**			4.80	0.74–31.2

*Bold: p < 0.05*.

Table 3 summarizes the ORs for the synergistic effect of unintended pregnancy and young maternal age on shaking. Infants of young mothers with unintended pregnancy were 2.77 times more likely to be shaken by their primary caregivers than those of older mothers with planned pregnancy (95% CI: 1.55–6.68). Infants of older mothers with unintended pregnancy were also more likely to be shaken than those with planned pregnancy (OR: 1.89, 95% CI: 1.13–3.15). In contrast, infants of young mothers with planned pregnancy were not at a significant risk of shaking. The odds ratio of young mothers with unintended pregnancy was not significantly different from that of older mothers with unintended pregnancy for shaking (*p* = 0.41). Therefore, there was no synergistic effect of unintended pregnancy and maternal age on shaking (*p* value for the interaction term, 0.55 (data not shown)).

**Table 3:** Odds ratios (OR) for interaction of unintended pregnancy and maternal age on shaking in 3- to 4-month-old infants.

		Prevalence of shaking (%)	Crude		Model 1		Model 2	
			OR	95% CI	OR	95% CI	OR	95% CI
Unintended pregnancy and maternal age	Intended, ≥25	1.6	Ref		Ref		Ref	
	Intended, <25	2.2	1.40	0.50-3.94	1.25	0.44–3.55	1.24	0.43–3.53
	Unintended, ≥25	2.9	**1.86**	**1.14–3.03**	**1.86**	**1.12–3.08**	**1.89**	**1.13–3.15**
	Unintended, <25	5.9	**3.94**	**1.74–8.93**	**2.99**	**1.28–7.03**	**2.77**	**1.15–6.68**

Late registration	No	1.8			Ref		Ref	
	Yes	3.7			1.76	0.96–3.22	1.68	0.91–3.11

Birth order	Subsequent child	1.5			Ref		Ref	
	First child	2.5			**1.70**	**1.07–2.72**	1.43	0.89–2.31

Returning to parents’ home for delivery	No	1.9			Ref		Ref	
	Yes	2.1			1.11	0.69–1.79	1.08	0.67–1.74
	Missing	4.2			1.98	0.58–6.75	2.26	0.65–7.84

Having someone who can help during pregnancy	No	2.1			Ref		Ref	
	Yes	2.0			1.18	0.36–3.84	1.34	0.41–4.46
	Missing	5.4			3.04	0.46–20.0	3.09	0.46–20.8

Worried/anxious during pregnancy	No	1.8			Ref		Ref	
	Yes	2.5			1.13	0.71–1.80	1.00	0.62–1.61

Depression during pregnancy	No	2.0			Ref		Ref	
	Yes	2.4			0.98	0.41–2.32	0.87	0.36–2.11

Crying	Not at all	1.0					Ref	
	Not so much	2.7					**2.71**	**1.56**–**4.70**
	Some extent to a lot	4.4					**4.17**	**2.31**–**7.49**

Economic hardship	No	1.9					Ref	
	Yes	2.3					0.79	0.37–1.68
	Missing	5.4					0.99	0.15–6.40

Postpartum depression by Edinburgh Postnatal Depression Scale score	≤8	1.8					Ref	
	≥9	4.8					**2.10**	**1.06**–**4.15**
	Missing	9.1					4.76	0.73–31.0

*Bold: p < 0.05*.

Table 4 summarizes the independent effects of unintended pregnancy and maternal age on smothering. Similar to the results for shaking, unintended pregnancy was strongly associated with smothering even after adjusting for prenatal and postpartum situation (OR: 2.57, 95% CI: 1.51–4.39), but there was no significant effect in the adjusted models.

**Table 4:** Odds ratios (OR) for unintended pregnancy and maternal age on smothering in 3- to 4-month-old infants.

		Prevalence of smothering (%)	Crude		Model 1		Model 2	
			OR	95% CI	OR	95% CI	OR	95% CI
Unintended pregnancy	No	1.1	Ref		Ref		Ref	
	Yes	2.8	**2.57**	**1.56–4.23**	**2.54**	**1.51–4.29**	**2.57**	**1.51–4.39**

Maternal age	≥25	1.4	Ref		Ref		Ref	
	<25	3.0	**2.12**	**1.04–4.32**	1.56	0.74–3.29	1.58	0.74–3.39

Late registration	No	1.6	Ref		Ref		Ref	
	Yes	1.3	0.84	0.34–2.11	0.68	0.27–1.75	0.54	0.21–1.42

Birth order	Subsequent child	0.8	Ref		Ref		Ref	
	First child	2.3	**2.84**	**1.62–4.96**	**2.66**	**1.49–4.72**	**2.43**	**1.35–4.37**

Returning to parents’ home for delivery	No	1.5	Ref		Ref		Ref	
	Yes	1.7	1.15	0.68–1.94	1.10	0.65–1.86	1.08	0.63–1.84

Having someone who can help during pregnancy	No	2.1	Ref		Ref		Ref	
	Yes	1.5	0.74	0.23–2.40	1.09	0.33–3.58	1.43	0.42–4.87

Worried/anxious during pregnancy	No	1.1	Ref		Ref		Ref
	Yes	2.3	**2.16**	**1.31–3.56**	1.62	0.96–2.74	1.31	0.76–2.25
	Missing	1.9	1.74	0.23–13.0	1.85	0.24–14.1	1.64	0.21–12.8

Depression during pregnancy	No	1.5	Ref		Ref		Ref	
	Yes	2.4	1.60	0.68–3.75	1.11	0.46–2.65	0.91	0.37–2.23

Crying	Not at all	0.7	Ref				Ref	
	Not so much	2.0	**2.69**	**1.45–4.99**			**2.23**	**1.18–4.19**
	Some extent to a lot	3.6	**4.97**	**2.62–9.43**			**3.60**	**1.87–6.97**

Economic hardship	No	1.5	Ref				Ref	
	Yes	1.8	1.19	0.54–2.64			0.58	0.25–1.37
	Missing	3.6	2.46	0.58–10.3			1.74	0.26–11.6

Postpartum depression by Edinburgh Postnatal Depression Scale score	≤8	1.2	Ref				Ref	
	≥9	7.2	**6.58**	**3.75–11.5**			**5.69**	**3.06–10.6**
	Missing	3.0	2.66	0.01–19.9			1.12	0.08–16.2

*Bold: p < 0.05*.

In models examining the synergistic effect of unintended pregnancy and maternal age on smothering (*p*-value for the interaction term, 0.09), infants of young mothers with unintended pregnancy were 5.61 times more likely to be smothered by their primary caregivers than those of older mothers with planned pregnancy (95% CI: 2.40–13.1) (Table 5). Infants of older mothers with unintended pregnancy were 2.12 times more likely to be smothered than those of older mothers with planned pregnancy (95% CI: 1.18–3.79). In contrast, young motherhood was not significantly associated with smothering when pregnancy was intended. The odds ratio of young mothers with unintended pregnancy was significantly different from that of older mothers with unintended pregnancy (*p* = 0.02), suggesting that the cooccurrence of unintended pregnancy and younger motherhood places a woman at a higher risk of smothering compared to older mothers with unintended pregnancy.

**Table 5:** Odds ratios (OR) for interaction of unintended pregnancy and maternal age on smothering in 3- to 4-month-old infants.

		Prevalence of smothering (%)	Crude		Model 1		Model 2	
			OR	95% CI	OR	95% CI	OR	95% CI
Unintended pregnancy and maternal age	Intended, ≥25	1.2	Ref		Ref		Ref	
	Intended, <25	0.5	0.47	0.06–3.44	0.44	0.06–3.25	0.42	0.06–3.16
	Unintended, ≥25	2.3	**2.03**	**1.17–3.51**	**2.13**	**1.21–3.76**	**2.12**	**1.18–3.79**
	Unintended, <25	6.7	**6.18**	**2.79–13.7**	**5.34**	**2.34–12.2**	**5.61**	**2.40–13.1**

Late registration	No	1.6			Ref		Ref	
	Yes	1.3			0.67	0.26–1.72	0.55	0.21–1.44

Birth order	Subsequent child	0.8			Ref		Ref	
	First child	2.3			**2.59**	**1.46–4.61**	**2.38**	**1.32–4.29**

Returning to parents’ home for delivery	No	1.5			Ref		Ref	
	Yes	1.7			1.10	0.65–1.87	1.08	0.63–1.85

Having someone who can help during pregnancy	No	2.1			Ref		Ref	
	Yes	1.5			1.08	0.33–3.55	1.45	0.42–5.01

Worried/anxious during pregnancy	No	1.1			Ref		Ref	
	Yes	2.3			1.62	0.96–2.74	1.32	0.77–2.28
	Missing	1.9			1.89	0.25–14.5	1.76	0.23–13.6

Depression during pregnancy	No	1.5			Ref		Ref	
	Yes	2.4			1.11	0.46–2.68	0.95	0.39–2.33

Crying	Not at all	0.7					Ref	
	Not so much	2.0					**2.22**	**1.18–4.18**
	Some extent to a lot	3.6					**3.57**	**1.84–6.91**

Economic hardship	No	1.5					Ref	
	Yes	1.8					0.58	0.25–1.38
	Missing	3.6					1.77	0.26–11.8

Postpartum depression by Edinburgh Postnatal Depression Scale score	≤8	1.2					Ref	
	≥9	7.2					**5.75**	**3.09–10.7**
	Missing	3.0					1.01	0.07–14.9

*Bold: p < 0.05*.

## Discussion

This is the first retrospective cohort study to examine the independent impact of unintended pregnancy on infant abuse by shaking and smothering, using large-scale survey data linking a 3- to 4-month health check questionnaire with pregnancy notification forms used in public health centers in Japan. We found that there is a synergistic effect of unintended pregnancy and young motherhood on smothering, which is independent of postpartum depression, perceived amount of infant crying, and subjective socioeconomic status, indicating unknown risk factors of shaking and smothering among this high-risk population.

The independent risk of infant abuse by unintended pregnancy is in line with previous evidence of associations between unintended pregnancy and other types of child maltreatment (15–17, 19). In general, unintended pregnancy may lead to unwanted marriage due to stereotypes of, or even stigma toward, single parents in Japanese culture (29), which could result in high levels of frustration during parenting. Further, it is not always easy for pregnant women to take maternity leave and childcare leave, with 22.3% of full-time female Japanese employees in 2015 reporting pregnancy and maternity discrimination at work (30), even though the Labor Standards Act and Child Care and Family Care Leave Act support the right to take maternity leave and child care leave, respectively. In the worst case scenario, some pregnant women may have to give up their careers, or quit their job, which may induce or exacerbate negative feelings about their pregnancy and increase parenting stress. Additionally, according to a previous study in Japan, mothers with unintended pregnancy were less likely to discuss and develop a plan for parenting (20). Unpreparedness can make parenting difficult and cause conflict in the family. Unintended pregnancy also increases parental stress associated with marital conflict and lack of knowledge about infant development and parenting (31). In particular, lack of knowledge about infant crying and SBS, which is more prevalent among Japanese parents than those in other countries (32), can cause stress and risk of abusive behaviors during the first few months of childrearing. Those with unintended pregnancy may even feel hostile and irritable toward their infants in these first few months (33), which can lead to abusive behaviors, such as shaking and smothering (34).

More importantly, we confirmed that there is a synergetic effect of unintended pregnancy and maternal age on smothering. That is, the risk of infant smothering is greatly increased when mothers are younger than 25 years old and their pregnancy is unintended, suggesting that young mothers have more difficulties dealing with unintended pregnancy than older mothers. This is consistent with the notion that there are factors present during the early postnatal months that make it stressful for young mothers to care for their infant, which can lead to an intent to kill or cause harm (9). Although reasons for smothering can vary from an urge to stop infant crying to an intention to end the infant’s life (34, 35), smothering shows a clear intention to kill or harm the infant compared to shaking, which indicates impetuous violence (9). The synergistic effect on smothering but not shaking was likely due to the fact that our target population comprised mostly mother, who are more likely to smother than shake their infants (9, 34). Further research should expand this study among new fathers.

Previous studies indicate that young maternal age (i.e., under 25 years old) is an independent risk factor of shaking and smothering (10, 36); however, we showed that when adjusted for unintended pregnancy, young maternal age was not significantly associated with shaking and smothering. Rather, the risk of shaking and smothering was greatest when unintended pregnancy and young maternal age were coincident. This highlights the importance of accounting for the comorbidity of unintended pregnancy and young motherhood in the prevention of infant abuse, especially smothering. Unintended pregnancy and maternal age are critical factors for the prevention of infant abuse in Japan because they can be identified in pregnancy notification forms. In Japan, mothers are encouraged to submit pregnancy notification forms to municipalities as soon as their pregnancy is confirmed so that they can receive Maternal and Child Health Handbooks and publically funded tickets for health checks. Pregnancy notification forms in Nagoya City inquire about expectant mothers’ feelings when notified of their pregnancy to understand mothers’ pregnancy intentions. According to our findings, we suggest that municipalities should utilize pregnancy notification forms to identify and support pregnant women with unintended pregnancy, especially young mothers, from the early stages of pregnancy. Further, it is also important for research to measure pregnancy intentions during pregnancy instead of using retrospective measures of pregnancy intentions to minimize recall bias (19).

Our study has several limitations. First, shaking and smothering behaviors were based on parental self-reports, not objective measurements. Unlike other countries, administrative data, court cases, and databases on shaking and smothering are not available in Japan. Although the extent of overlap between self-reported cases and AHT/SBS hospital data is unknown, previous studies have used self-administered questionnaires to assess the prevalence of shaking and smothering (10, 11, 37, 38). Second, previous occurrence of shaking and smothering toward other children of the caregivers was not examined in our study. That is, it remains unclear whether shaking and smothering behaviors are influenced by maternal characteristics *per se*, such as age and hostility toward children, or relationships between the caregiver and their infant. For example, some caregivers may shake or smother their infant due to a relationship with that particular infant, while others may shake or smother their infant and his/her sibling(s) due to the caregiver’s own characteristics. Further research is needed to distinguish between these factors. Third, we did not examine the pregnancy intentions of fathers. Guterman (19) argued that fathers’ pregnancy intentions were as important as that of mothers’, and were associated with abusive behaviors. Further, pregnancy intentions of parents are interactive, and disagreement in pregnancy intentions can affect relationships (19). Future research should include fathers’ pregnancy intentions to better understand the association between unintended pregnancy and child maltreatment, especially for shaking, for which fathers are the main perpetrators (9, 39, 40). Fourth, our study did not examine any changes to pregnancy intentions during pregnancy. Since pregnancy intentions are subjective and affected by social circumstances, they can change in either direction over time (16). The risk of those who may have developed negative feelings later in their pregnancy was not examined. It is important to develop a system that can find and support such pregnant women, such as health checks, home visitation, and pregnancy counseling services. Fifth, our definition of unintended pregnancy may not be ideal. Although we defined unintended pregnancy based both on the responses to questions about expectant mothers’ feelings when notified of their pregnancy and the prevalence of shaking and smothering, further investigation using direct assessment of pregnancy intentions is warranted. Sixth, only depression was measured as a maternal mental health issue. Other mental health issues, such as hostility and anxiety, may be associated with maternal age, pregnancy intentions, and infant child abuse. These mental health issues prior to pregnancy should be considered in future studies because unmeasured cofounders can cause bias. Finally, those who did not submit pregnancy notification forms were omitted in this study, including those who translocated from another city and those who experienced precipitate delivery without perinatal care. Since women who undergo delivery without prenatal care can be at a higher risk of child abuse (41, 42), it is likely that we underestimated our findings. Therefore, public health services, including pregnancy counseling services, are essential for supporting those who have not reported their pregnancy to municipalities in the aims of infant abuse prevention.

In conclusion, young motherhood and unintended pregnancy place women at a greater risk of shaking and smothering after controlling for prenatal situations assessed in pregnancy notification forms and postpartum situations assessed in a questionnaire at 3- to 4-month health check. Prevention programs should target young pregnant women with unintended pregnancy. Further studies are needed to examine the long-term effect of unintended pregnancy and maternal age on maltreatment among toddlers.

## Ethics Statement

This study was approved by the institutional review board at the National Center for Child Health and Development (Study ID: 716) and Tokyo Medical and Dental University (Study ID: M2016-287) in Tokyo, Japan. We informed participants of the linking of the questionnaire and pregnancy form *a priori*, and the return of a completed survey was considered consent to participate in the study.

## Author Contributions

AI and TF conceived the study; TF collected the data; AI analyzed and wrote the first draft; and AI and TF finalized the manuscript. Both authors approved the final version of the manuscript.

## Conflict of Interest Statement

The authors declare that the research was conducted in the absence of any commercial or financial relationships that could be construed as a potential conflict of interest.
